# Transesterification Synthesis of Chloramphenicol Esters with the Lipase from *Bacillus amyloliquefaciens*

**DOI:** 10.3390/molecules22091523

**Published:** 2017-09-19

**Authors:** Fengying Dong, Lingmeng Li, Lin Lin, Dannong He, Jingwen Chen, Wei Wei, Dongzhi Wei

**Affiliations:** 1 State Key Laboratory of Bioreactor Engineering, Newworld Institute of Biotechnology, East China University of Science and Technology, 130 Meilong Road, Shanghai 200237, China; fengyingdong16@163.com (F.D.); 18993965077@163.com (L.L.); 2 Research Laboratory for Functional Nanomaterial, National Engineering Research Center for Nanotechnology, Shanghai 200241, China; linlin21023@163.com (L.L.); hdnbill@sh163.net (D.H.); 3 Department of Pathology, Microbiology and Immunology, School of medicine, University of South Carolina, 6311 Garners Ferry Rd., Columbia, SC 29209, USA; linye@mailbox.sc.edu

**Keywords:** enzymatic catalysis, regioselectivity, chloramphenicol esters, green chemistry, *Bacillus amyloliquefaciens*

## Abstract

This work presents a synthetic route to produce chloramphenicol esters by taking advantage the high enantio- and regio-selectivity of lipases. A series of chloramphenicol esters were synthesized using chloramphenicol, acyl donors of different carbon chain length and lipase Lip_BA_ (lipase cloned from *Bacillus amyloliquefaciens*). Among acyl donors with different carbon chain lengths, vinyl propionate was found to be the best. The influences of different organic solvents, reaction temperature, reaction time, enzyme loading and water content on the synthesis of the chloramphenicol esters were studied. The synthesis of chloramphenicol propionate (0.25 M) with 4.0 g L^−1^ of Lip_BA_ loading gave a conversion of ~98% and a purity of ~99% within 8 h at 50 °C in 1,4-dioxane as solvent. The optimum mole ratio of vinyl propionate to chloramphenicol was increased to 5:1. This is the first report of *B. amyloliquefaciens* lipase being used in chloramphenicol ester synthesis and a detailed study of the synthesis of chloramphenicol propionate using this reaction. The high enzyme activity and selectivity make lipase Lip_BA_ an attractive catalyst for green chemical synthesis of molecules with complex structures.

## 1. Introduction

In recent years, enzymes have been applied to synthesize chloramphenicol derivatives, which has aroused widespread interest because of their high activity, moderate catalytic conditions, and environmental friendliness. This due to the fact that the enzymatic approach avoids the need for protection and deprotection procedures to discriminate between the different available hydroxyl groups in chloramphenicol, as chloramphenicol substituents on the primary hydroxyl group are rapidly hydrolyzed in vivo to the biologically active drug [1]. In addition, the separation steps to remove impurities that have close physical and chemical properties to the target compound results in lower final conversion, higher costs, time-and energy-consumption and excess discharge of waste. In this sense, enzymatic catalysis with its high enantio- and regioselectivity is attractive for the green synthesis of chemical compounds.

Chloramphenicol is a natural antibiotic with a wide spectrum of antimicrobial activity against Gram-positive and Gram-negative bacteria [2,3,4]. Recently, chloramphenicol has been administered in increasing dosages due to the increased incidence of antibiotic resistance [5]. Unfortunately, it can also produce liver and kidney function inhibition, grey baby syndrome, aplastic anemia and so on. Meanwhile the bitterness of chloramphenicol cannot be accepted by most people [6], so different chloramphenicol derivatives have been produced minimize this bitterness [1] such as chloramphenicol succinate or chloramphenicol palmitate esters produced by means of regio- or stereoselective chemical [7] and enzymatic methods [8]. Among all catalytic enzymes, lipases catalyze transesterification reactions on hydroxyl groups with high regioselectivity and mild reaction conditions [9,10,11]. Daugs et al. reported the lipase-mediated esterification of chloramphenicol palmitate in toluene and DMF [12]. Bizerra et al. reported that the *Candida antarctica* lipase type B (CAL-B) catalyzed the synthesis of chloramphenicol palmitate (0.15 M) to reach 99% conversion in 24 h at 50 °C [8]. Using the non-imprinted lipase nanogel and the lipase from *Thermomyces lanuginosus* [13], Wang et al. produced chloramphenicol palmitate with a conversion of 99% within 20 h at 20 °C. Ottolina et al. reported lipase G was the best biocatalytic agent, giving an excellent conversion to the corresponding esters at 45 °C after 24–72 h [14]. Further, Ottolina et al. reported the lipase-mediated esterification of chloramphenicol for the synthesis of several derivatives in anhydrous acetone to explore the effect of different trifluoroethyl esters. In this research most studies have focused on the synthesis of a single chloramphenicol ester, performed at an undesirable low temperature, with low substrate concentration and long reaction times.

Previously, our group reported lipase Lip_BA_ (accession number: KF040967) cloned from *Bacillus amyloliquefaciens* and the detailed enzymatic properties of the recombinant enzyme [15]. Furthermore, in our previous report, this enzyme was used in biocatalysis and cinnamyl esters were synthesized using lipase (Lip_BA_) through the transesterification route in a non-aqueous system with vinyl propionate as the best acyl donor [16]. In this study, we have developed a chloramphenicol ester synthesis by using different carbon chain length acyl donors [17]. Among the acyl donors with different carbon chain lengths, vinyl propionate was chosen to act as the best acyl donor. Through single factor experiments, the enzymatic synthesis of chloramphenicol propionate ester was studied in detail for the first time, with different factors affecting the conversion efficiency. Furthermore, the transesterification reaction yield at high substrate concentrations (0.25 M) was the highest, and the reaction time (8 h) was the shortest at this concentration (0.25 M) in the previous reported literature. Using 0.25 M substrate concentrations, the chloramphenicol propionate provided excellent yield (98%) and purity (99%) within 8 h at 50 °C in 1,4-dioxane as solvent. Owing to the enzymatic properties such as high substrate concentration, high conversion rate, high product yield and purity, this lipase has potential value in industrial applications, particularly for chloramphenicol propionate synthesis.

## 2. Results and Discussion

### 2.1. Synthesis of Chloramphenicol Propionate Esters

**Figure 1:**
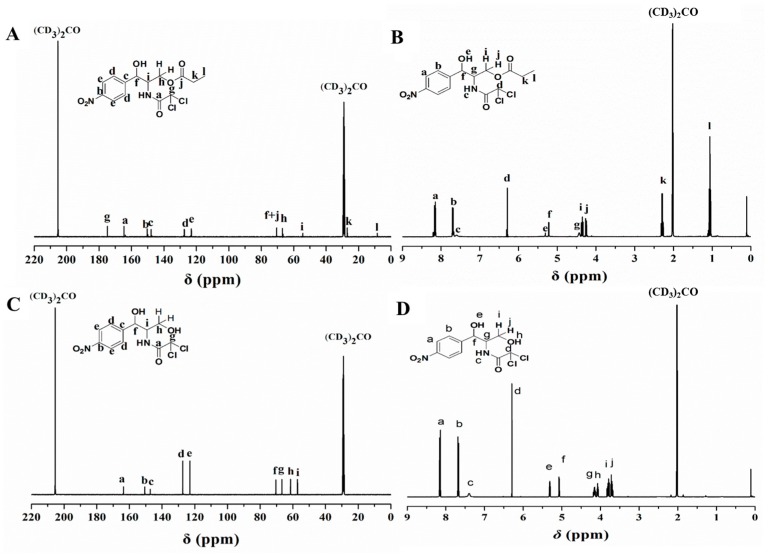
NMR spectra of chloramphenicol propionate ester and chloramphenicol. (**A**) ^13^C-NMR spectrum of chloramphenicol propionate ester in (CD_3_)_2_CO; (**B**) ^1^H-NMR spectrum of chloramphenicol propionate ester in (CD_3_)_2_CO; (**C**) ^13^C-NMR spectrum of chloramphenicol in (CD_3_)_2_CO; (**D**) ^1^H-NMR spectrum of chloramphenicol in (CD_3_)_2_CO.

The transesterification reaction of chloramphenicol with vinyl propionate was performed using Lip_BA_ in 1,4-dioxane. The products were purified by silica gel chromatography and characterized by ^13^C-NMR and ^1^H-NMR analysis. As Yoshimoto [18] established, acylation of a sugar hydroxyl group results in a downfield shift of the peak corresponding to the *O*-acylated carbon and an upfield shift of the peak corresponding to the neighboring carbon. Characterization of the chloramphenicol ester by ^13^C-NMR revealed that the signals for R_1_ of the chloramphenicol ester were shifted downfield and the C-2 positions shifted upfield (Figure 1A), compared with chloramphenicol (Figure 1C), indicating chloramphenicol was substituted at the R_1_ position. Almost no shifting of the peak corresponding to the secondary alcohol in the product was observed, compared with chloramphenicol (Figure 1C). Furthermore, the ^1^H-NMR spectrum showed the two hydrogens on the methylene groups of the chloramphenicol ester groups as characteristic peaks i and j at 4.36 and 4.24 ppm (Figure 1B), respectively, with a significant downfield shift, compared with 3.80, 3.71 ppm for chloramphenicol (Figure 1D). ^1^H-NMR also showed that the hydrogen on the methine group and secondary alcohol of the chloramphenicol ester groups give the characteristic peaks f and e at 5.267 and 5.368 ppm (Figure 1B), and these undergo a minor shift, compared with chloramphenicol at 5.137 and 5.373 ppm (Figure 1D).^1^H-NMR also confirmed the regioselective acylation at the primary hydroxyl group (R_1_). These results all confirm that Lip_BA_ showed effective regioselectivity in the transesterification of chloramphenicol with vinyl propionate.

### 2.2. Transesterification of Chloramphenicol with Different Acyl Donors

**Figure 2:**
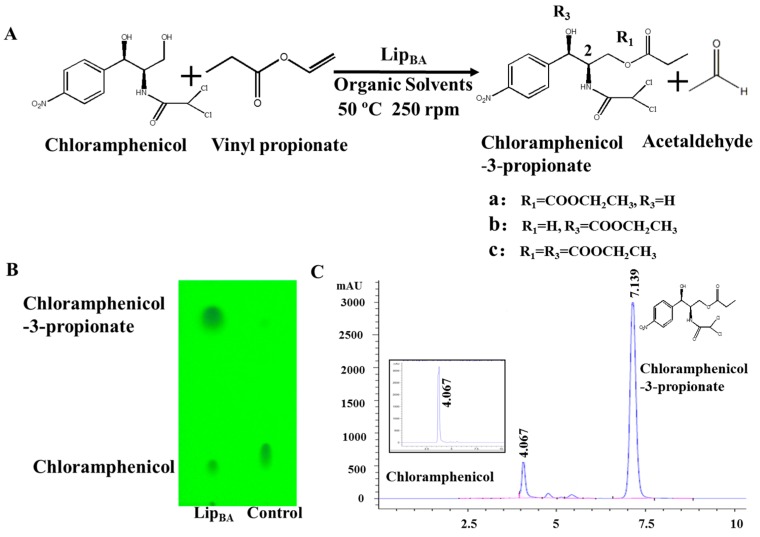
Lipase-mediated esterification of chloramphenicol using vinyl propionate in organic solvents. (**A**) Preparation of chloramphenicol propionate through transesterification; (**B**) TLC analysis of product; (**C**) Chromatogram of chloramphenicol and chloramphenicol-3-propionate. The assay conditions were described in material and methods.

Single factor experiments were carried out in our study. Firstly, the transesterification of chloramphenicol with different acyl donors was investigated. These were vinyl acetate, vinyl propionate, vinyl butyrate, vinyl neononanoate, vinyl decanoate and vinyl laurate, respectively. Recent experimental studies on chloramphenicol ester synthesis by enzymatic catalysis are listed in Table 1. The preparation of chloramphenicol esters through transesterification is shown in Figure 2A. These data revealed that lipase can be applied in chloramphenicol ester synthesis with acyl donors as transesterification substrates. Among the different acyl donors vinyl propionate led to quantitative and high conversion (81%) to the corresponding chloramphenicol ester under the same reaction conditions (Table 2, reaction conditions: enzyme loading 4.0 g L^−1^; chloramphenicol 0.25 M in ethanol; acyl donor/chloramphenicol = 5:1; T = 50 °C; 4 h and 200 rpm). This trend showes that Lip_BA_ gives higher conversion with short chain rather than medium and long chain donors. Chloramphenicol gave a lower conversion (≤55%) to the esters using other acyl donors because of the poorer reactivity of these acyl donors. It should be noted that the diacylated compounds b (R_3_ = COOCH_2_CH_3_, R_1_ = H, Figure 2) and c (R_1_ = R_3_ = COOCH_2_CH_3_, Figure 2) were not detected after 8 h. These experimental results were consistent with the reports from Bizerra and Daugs [8,12]. This effect can be explained because diacylated compound b and c could not correctly fit in the active site of Lip_BA_. The above achievements suggest Lip_BA_ lipase is an ideal catalyst for the regioselective acylation of chloramphenicol, showing an excellent selectivity for the acylation of the primary alcohol [8,17]. The molar ratio of chloramphenicol to vinyl propionate had a significant effect on the conversion of the product. A poor conversion (69%) was obtained at the molar ratio of chloramphenicol to vinyl propionate of 1:1. When the molar ratio of vinyl propionate to chloramphenicol was increased to 5:1, the conversion of chloramphenicol was 81% (Table 2). There was no significant further increase in the product yield with a further increase in the amount of vinyl propionate used, therefore the molar ratio of vinyl propionate to chloramphenicol used was 5:1.

### 2.3. Effect of Different Solvents

**Figure 3:**
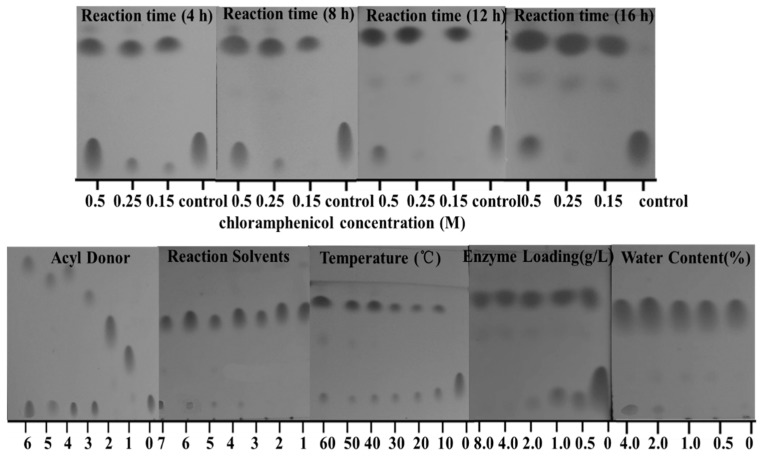
Effect of the reaction conditions on the conversion of chloramphenicol. Reaction conditions: Chloramphenicol concentration: 0.15, 0.25, 0.5 M; acyl donor: 0: control, 1: vinyl acetate, 2: vinyl propionate, 3: acetone, 4: vinyl neononanoate, 5: vinyl decanoate, 6: vinyl laurate; Reaction solvent: 1: ethanol, 2: 1,4-dioxane, 3: acetone, 4: acetonitrile, 5: tetrahydrofuran, 6: dichloromethane, 7: toluene; temperature: 0: control, T = 10, 20, 30, 40, 50, 60 °C; enzyme loading = 0, 0.5, 1.0, 2.0, 4.0, 8.0 g L^−1^; water content = 0%, 0.5%, 1.0%, 2.0%, 4.0%; 8 h and 200 rpm.

The effect of solvents on enzymatic reactions is critical for a non-aqueous medium [20]. The solvent affects the catalytic power of enzymes by changing the three-dimensional conformation of proteins, and therefore significantly alters the conversion [21]. It is reported that enzymes had a better stability and compatibility in non-polar solvents than in polar solvents, due to the fact polar solvents with high *E_T_* values show bad compatibility with enzyme molecules that leads to reduced activity [22]. In this study, the polarity of the reaction medium was selected according to the empirical polarity parameter *E*_T_(30) described by Reichardt for pure solvents and adapted by Castillo et al. [23,24,25]. *E*_T_(30) takes into account solvent–solute interactions at the molecular level [26]. The effect of different media (toluene, dichloromethane, tertrahydrofuran, 1,4-dioxane, acetonitrile, acetone and ethanol) ranging from *E*_T_(30) 33.9 (non-polar) to *E*_T_(30) 51.9 (polar) were studied with vinyl propionate as acyl donor (Table 3). The reaction conditions were as follows: enzyme loading 4.0 g L^−1^; chloramphenicol 0.25 M in different organic solvents; vinyl propionate/chloramphenicol = 5:1; T = 50 °C; 4 h and 200 rpm. From the results, 1,4-dioxane was the best organic solvent and gave a highest conversion (89%) of chloramphenicol ester (chloramphenicol propionate) (Table 3, Figure 3). The results in Table 3 indicate that Lip_BA_ had better operational stability and tolerance (67% residual enzyme activity after 12 h) in 1,4-dioxane. Furthermore, both chloramphenicol and vinyl propionate had good solubility and compatibility in 1,4-dioxane. This was favorable to the organic reactants near the active sites of catalyst (Lip_BA_), which effectively promoted the occurrence of the reaction. Therefore 1,4-dioxane was chosen as the optimum reaction solvent. In this study, under the same reaction conditions, 0.15 M chloramphenicol can be fully converted into chloramphenicol propionate. 0.25 M chloramphenicol can be fully dissolved in 1,4-dioxane and gave a higher conversion compared with 0.5 M (Figure 3), so 0.25 M was the ideal concentration for further reaction condition optimization.

### 2.4. Effect of Reaction Temperature

Enzyme stability decreased with the increase of temperature out of a certain range. The effect of reaction temperature on esterification was studied at 20, 30, 40, 50 and 60 °C for 4 h. The reaction conditions were as follows: enzyme loading 4.0 g L^−1^; chloramphenicol 0.25 M in 1,4-dioxane; vinyl propionate/chloramphenicol = 5:1; reaction time 4 h and 200 rpm. The results indicated that the conversion (91%) and purity (99%) reached their maximum value at 50 °C (Table 3, Figure 3). With further increasing temperature, the conversion and purity declined gradually. The results revealed that the increasing temperature facilitated the nucleophilic substitution reaction when the temperature was within a certain range, thus enhancing the reaction rate. However, a higher temperature gradually inactivated the enzyme and degraded the product due to the poor product stability. On the other hand, it was also possible that the elevated temperature promoted reactivity of the secondary alcohol group and thus brought a negative impact on the lipase efficient [27].

### 2.5. Effect of Reaction Time

Reaction time studies indicated the performance of the enzyme as the reaction progressed. Furthermore, the reaction time helped to determine the shortest time necessary to obtain a high conversion. The effect of reaction time on esterification was studied at 4, 6, 12 and 16 h. The reaction conditions were as follows: enzyme loading 4.0 g L^−1^; chloramphenicol 0.25 M in 1,4-dioxane; vinyl propionate/chloramphenicol = 5:1; reaction temperature 50 °C. As shown in Table 3, when the time was increased to 8 h, chloramphenicol was almost fully converted to chloramphenicol propionate with a conversion of 98% and a purity over 99% after 8 h reaction at 50 °C (Table 3, Figure 3). Increasing the reaction time up to 16 h led to a decrease in both conversion (92%) and purity (85%). This might be due to the poor stability of the chloramphenicol propionate and the elevated reactivity of the secondary alcohol group at the longer time.

### 2.6. Effect of Enzyme Loading

**Figure 4:**
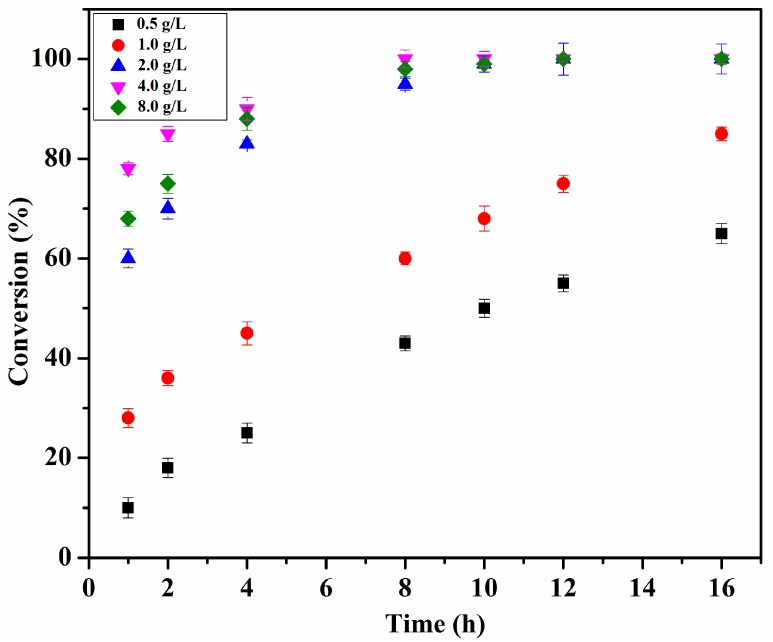
Enzyme loading effect on the conversion of chloramphenicol. Reaction conditions: chloramphenicol 0.25 M in 1,4-dioxane; vinyl propionate/chloramphenicol = 5:1; T = 50 °C; 8 h and 200 rpm.

The enzyme loading is crucial for any bioconversion process. Thus, the effect of enzyme loading was studied for different enzyme concentrations such as 0.5, 1.0, 2.0, 4.0 and 8.0 g L^−1^ of the reaction volume. The reaction conditions were as follows: chloramphenicol 0.25 M in 1,4-dioxane; vinyl propionate/chloramphenicol = 5:1; T = 50 °C; 8 h and 200 rpm. As shown in Figure 4, the conversion increased with Lip_BA_ loading up to 4.0 g L^−1^. Enzyme loading over 4.0 g L^−1^ not only led to no improvement in the conversion rate, but also caused lower conversion and purity (Figure 4). This may be due to the formation of enzyme aggregates which led to a drop in the exposed surface area of the catalyst to the reactants and accordingly a decreased mass transfer. Enzyme particles present on the inner side of aggregates were not available to react with the substrate which reduced the overall interfacial area [20,28]. These data suggested that 4.0 g L^−1^ of enzyme loading was optimal for the conversion and purity.

### 2.7. Influence of the Water Content

**Figure 5:**
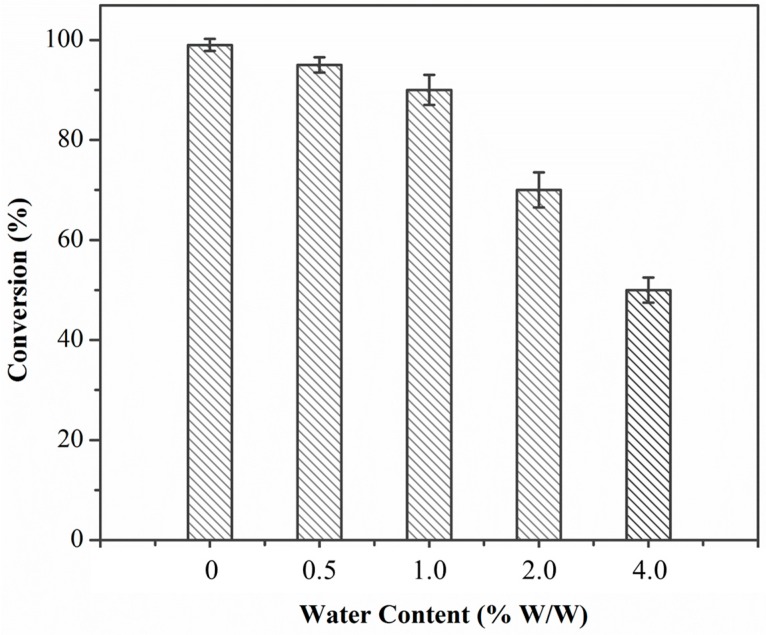
Water content effect on the conversion of chloramphenicol. Reaction conditions: enzyme loading 4.0 g L^−1^; chloramphenicol 0.25 M in 1,4-dioxane; vinyl propionate/chloramphenicol = 5:1; T = 50 °C; 8 h and 200 rpm.

Water content is essential in any organic biosynthesis for enzyme flexibility and for optimal catalytic activity [29]. The water content effect on Lip_BA_ initial activity was examined using water concentrations ranging from 0% to 4.0% (*w*/*w*) in 1,4-dioxane. As shown in Figure 5, the less water in the medium, the more the transesterification reaction was favored. The synthesis activity of chloramphenicol propionate with Lip_BA_ was significantly reduced at water contents up to 2.0% (*w*/*w*), which was consistent with some previous studies indicating that the synthetic activity of most lipases was optimal at relatively low water content in organic systems (typically below 1% (*w*/*w*)) [22]. In this reaction, the reduction of synthesis activity would be due to a larger amount of water in the organic medium. Water would reduce the solubility and the diffusibility of the substrates and the ester product. Therefore, the conversion would be decreased with the increasing water content [15,17,30].

Finally, according to the single factor experiment results, the optimum reaction condition was as follows: acyl donor: vinyl propionate; organic solvent: 1,4-dioxane; reaction temperature: 50 °C; reaction time: 8 h; enzyme loading: 4.0 g L^−1^; substrate concentration: chloramphenicol 0.25 M, vinyl propionate/chloramphenicol = 5:1. Under this condition, chloramphenicol was almost fully converted to chloramphenicol propionate with a conversion of 98% and a purity over 99%.

## 3. Materials and Methods

### 3.1. Materials

The lipase Lip_BA_ was obtained from *B. amyloliquefaciens* Nsic8 as previously reported in our research team, and the specific activity was 1750 ± 153 U mg^−1^ [15]. One unit (U) of lipase activity was defined as the amount of enzyme required to produce 1 μmol of product per min under the defined assay conditions [31]. Chloramphenicol and various vinyl esters were purchased from Sigma-Aldrich (St. Louis, MO, USA). 1,4-Dioxane, ethyl acetate and other chemicals were analytical reagent grade and were used without further purification.

### 3.2. Enzymatic Synthesis of Cinnamyl Acetate and Single Factor Experiment

The whole-cell catalyst containing lipase Lip_BA_ were centrifuged, washed once with 100 mM Tris–HCl (pH 8.0), and then lyophilized by vacuum freezing. The transesterification reaction was carried out in a 50 mL Eppendorf tube and 1,4-dioxane was used as solvent. To determine the optimum reaction parameters in the chloramphenicol ester synthesis reaction, single factor experiments were carried out in this study. Firstly, different acyl donors (vinyl acetate, vinyl propionate, vinyl butyrate, vinyl decanoate, vinyl laurate and vinyl palmitate) were used. Secondly, the effect of different organic solvents (toluene, dichloromethane, tertrahydrofuran, 1,4-dioxane, acetonitrile, acetone and ethanol) with varying *E*_T_(30) values, were studied. Thirdly, the effect of reaction temperature was studied at 20, 30, 40, 50 and 60 °C. The reactor was shaken at 200 rpm and immersed in a thermostatic water bath to keep the system within ±2 °C of the desired temperature. Fourthly, the effect of reaction time (4, 8, 12 and 16 h) was detected. Fifthly, the enzyme loading was studied in the range from 0.5 to 8.0 g L^−1^. The effect of water concentration (ranging from 0% to 4.0% (*w*/*w*)) was analysis. All experiments were performed under a nitrogen atmosphere. Aliquots were regularly analyzed by HPLC and the reaction was stopped after complete consumption of the starting material. Finally the enzyme was filtered off. The solvent was evaporated under reduced pressure to get the total products. A parallel reaction under the same conditions without the addition of the enzyme was used as a control. All experiments were performed three times.

### 3.3. Analysis Method

The progress of reactions was by TLC using a ZF-1 UV analyzer to visualize the spots (254 nm) [19]. The prepared samples were spotted onto the silica gel TLC plate, and then the plate was placed in a chamber containing a solvent system of *n*-hexane and ethyl acetate (7:3). After the plate was dried, the products bands were dyed by solid iodine (Figure 2B). The conversion was calculated by HPLC (Waters 2690 Separations Module; Waters 2487 UV/Vis Dual Absorbance Detector; Waters, Milford, MA, USA; readings were made at 280 nm, Figure 2C) using a silica gel column (Waters Symmetry TM, C8, 3.9 × 150 mm) eluted with methanol/water (70:30), flow rate, 1 mL/min [17]. The crude reaction product was purified by silica gel chromatography and characterized by NMR analysis. The position of acylation in the enzyme-prepared chloramphenicol esters was established by ^1^H-NMR (400 MHz, (CD_3_)_2_CO) and ^13^C-NMR (101 MHz, (CD_3_)_2_CO) on a RESONANCE ECZ 400S spectrometer (JEOL Resonance Inc., Tokyo, Japan). Deuterated acetone was used as solvent; trimethylsilane was used as an internal reference.

## 4. Conclusions

In this study, the effects of various parameters on the lipase (Lip_BA_) catalyzed chloramphenicol transesterification with different acyl donor in organic solvents were studied. Among different acyl donors, vinyl propionate had the highest conversion efficiency in 1,4-dioxane as solvent. Overall a conversion of 98% and a purity of 99% were achieved in 8 h using 4.0 g L^−1^ of catalyst at 50 °C. The optimum mole ratio of vinyl propionate to chloramphenicol was 5:1. In the present work, we have shown that lipase Lip_BA_ could easily be used as an efficient whole-cell biocatalyst for the synthesis of short-chain chloramphenicol esters. In organic media, the Lip_BA_ showed high conversion and regio-selectivity. Furthermore, the biocatalyst displayed high versatility toward short-chain precursors typical of chloramphenicol esters. These results demonstrate that lipase Lip_BA_ with a high catalytic activity and regio- and stereoselectivity, holds great promise for the green synthesis of chemicals with complex structures in organic media.

## Figures and Tables

**Table 1:** Recent experimental studies in chloramphenicol esters synthesis by enzymatic catalysis.

Chloramphenicol Esters	Enzyme	Resource	Concentration	t (h)	Solvent	Reference
Chloramphenicol acetate	CAL-B	*C. antarctica lipase*	0.15 M	40	1,4-dioxane,	[8]
	CAT	*S. aureus*	0.0015 M	12	phosphate buffer	[19]
Chloramphenicol propionate	CAL-B	*C. antarctica lipase*	0.15 M	6	1,4-dioxane,	[8]
	CAT	*S. aureus*	0.0015 M	12	phosphate buffer	[19]
Chloramphenicol butyrate	CAT	*S. aureus*	0.0015 M	12	phosphate buffer	[19]
Chloramphenicol succinate	NR	--	--	--	--	--
Chloramphenicol pivalate	NR	--	--	--	--	--
Chloramphenicol decanoate	NR	--	--	--	--	--
Chlor5amphenicol laurate	CAL-B	*C. antarctica lipase*	0.15 M	24	1,4-dioxane	[8]
Chloramphenicol cinnamate	NR	--	--	--	--	--
Chloramphenicol palmitate	CAL-B	*C. antarctica lipase*	0.15 M	24	1,4-dioxane	[8]
	nanogel	*T. lanuginosus*	0.15 M	20	acetonitrile	[13]
	lipase	--	70 mM	120	toluene	[17]
Chloramphenicol propionate	Lip_BA_	*B.amyloliquefaciens*	0.25 M	8	1,4-dioxane	--

NR: not reported.

**Table 2:** The acyl donors’ effect on the yield of chloramphenicol. Reaction conditions: enzyme loading 4.0 g L^−1^; chloramphenicol 0.25 M in ethanol; acyl donor/chloramphenicol = 5:1; T = 50 °C; 4 h and 200 rpm.

Compound	Chemical Structure	Ratio	Time (h)	Conversion ^a^
Vinyl acetate	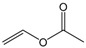	5:1	4	55%
Vinyl propionate	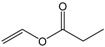	5:1	4	81%
Vinyl butyrate	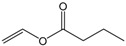	5:1	4	30%
Vinyl neononanoate	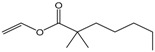	5:1	4	23%
Vinyl decanoate	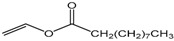	5:1	4	20%
Vinyl laurate	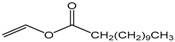	5:1	4	8%
Vinyl propionate	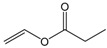	5:1	4	69%
Vinyl propionate	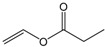	10:1	4	82%
Vinyl propionate	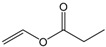	15:1	4	84%

^a^ Determined by HPLC and referred to the initial concentration of chloramphenicol. Conversions determined by HPLC after 4 h of reaction.

**Table 3:** Single experiments in the chloramphenicol ester synthesis reaction.

Factor	Solvent	*E*_T_(30)	Temperature	Time	Conversion ^b^ (%)	Purity (%)
Solvent	Control	-	40 °C	4 h	0	0
	Toluene	33.9	40 °C	4 h	50 ± 1.2	50 ± 0.7
	1,4-Dioxane	36.0	40 °C	4 h	89 ± 2.1	95 ± 2.5
	THF	37.4	40 °C	4 h	70 ± 2.3	85 ± 2.6
	Dichloromethane	40.7	40 °C	4 h	60 ± 1.5	70 ± 1.5
	Acetone	42.2	40 °C	4 h	72 ± 0.7	75 ± 1.2
	Acetonitrile	45.6	40 °C	4 h	80 ± 1.4	85 ± 0.9
	Ethanol	51.9	40 °C	4 h	81 ± 0.9	87 ± 0.5
Temperature	1,4-Dioxane	36.0	20 °C	4 h	85 ± 1.7	93 ± 1.3
	1,4-Dioxane	36.0	30 °C	4 h	87 ± 2.3	95 ± 2.1
	1,4-Dioxane	36.0	40 °C	4 h	89 ± 1.5	95 ± 2.8
	1,4-Dioxane	36.0	50 °C	4 h	91 ± 2.2	99 ± 1.9
	1,4-Dioxane	36.0	60 °C	4 h	90 ± 1.9	93 ± 2.4
Time	1,4-Dioxane	36.0	50 °C	8 h	98 ± 0.6	99 ± 1.5
	1,4-Dioxane	36.0	50 °C	12 h	96 ± 1.8	90 ± 1.2
	1,4-Dioxane	36.0	50 °C	16 h	92 ± 2.1	85 ± 0.7

^b^ All reactions were performed in 10 mL 1,4-dioxane containing 4.0 g L^−1^ Lip_BA_, 0.25 M chloramphenicol, and 1.25 M Vinyl propionate. Conversion was determined by HPLC and referred to the initial concentration of chloramphenicol. Purity was determined by flash chromatography.
